# Transcriptomic Insights into the Insect Immune Response to Nematode Infection

**DOI:** 10.3390/genes12020202

**Published:** 2021-01-30

**Authors:** Ioannis Eleftherianos, Christa Heryanto

**Affiliations:** Infection and Innate Immunity Lab, Department of Biological Sciences, Institute for Biomedical Sciences, The George Washington University, Washington, DC 20052, USA; christah@gwu.edu

**Keywords:** insect, nematodes, infection, immunity, transcriptomics

## Abstract

Insects in nature interact with a wide variety of microbial enemies including nematodes. These include entomopathogenic nematodes that contain mutualistic bacteria and together are able to infect a broad range of insects in order to complete their life cycle and multiply, filarial nematodes which are vectored by mosquitoes, and other parasitic nematodes. Entomopathogenic nematodes are commonly used in biological control practices and they form excellent research tools for understanding the genetic and functional bases of nematode pathogenicity and insect anti-nematode immunity. In addition, clarifying the mechanism of transmission of filarial nematodes by mosquitoes is critical for devising strategies to reduce disease transmission in humans. In all cases and in order to achieve these goals, it is vital to determine the number and type of insect host genes which are differentially regulated during infection and encode factors with anti-nematode properties. In this respect, the use of transcriptomic approaches has proven a key step for the identification of insect molecules with anti-nematode activity. Here, we review the progress in the field of transcriptomics that deals with the insect response to nematode infection. This information is important because it will expose conserved pathways of anti-nematode immunity in humans.

## 1. Introduction

Insects occupy different natural habitats and face a constant battle for survival when confronting microbial challenges. They respond to microbial invaders by activating their innate immune system, which involves the detection of pathogens through pattern recognition receptors, the induction of intracellular immune signaling pathways, and the regulation of humoral and cellular immune mechanisms [1,2,3]. Insect host defenses include hemocyte-related functions and the production of antimicrobial peptides and other effector molecules, which are secreted into the hemolymph [4,5,6]. Understanding immune reactions in insects has been facilitated by the use of insect models that permit elegant genetic manipulations in the laboratory, but significant discoveries have also been made in insects of agricultural or medical importance [7,8,9,10].

Distinct attention has been given recently to the interaction between insect hosts and nematode parasites. Nematodes employ multiple strategies to disrupt the activation and normal function of insect physiological responses, many of which participate in the regulation of innate immune reactions [11]. For instance, the entomopathogenic nematodes belonging to the genera *Heterorhabditis* and *Steinernema* are inhabited by the Gram-negative bacteria *Photorhabdus* and *Xenorhabdus*, respectively, and they are capable of entering insects that mainly live in the soil in order to reproduce by feeding on various tissues before they search for other victims to infect [12]. In addition, filarial nematodes, which cause important diseases in humans, are transmitted primarily by mosquitos that mount antimicrobial peptide and melanotic encapsulation responses against the parasites [13]. In addition, other parasitic nematodes are able to infect diverse insects, like *Howardula* nematodes that infect mycophagus *Drosophila* species [14].

Elucidating the molecular interactions between insects and parasitic nematodes as well as the genetic basis of the insect response to nematode infection require methods that facilitate characterization of the global gene expression changes that occur at the organismal and tissue/cell-specific levels. Here, we review the recent progress on the implementation of various transcriptomic approaches into the identification and characterization of gene expression regulation and signaling pathway activation, which modulate anti-nematode processes in insects. Such information is essential for the discovery of molecules with anti-nematode activity.

## 2. Insect Transcriptomic Response to Entomopathogenic Nematodes 

The majority of studies on the transcriptomic response of insect to entomopathogenic nematodes have been performed in *Drosophila melanogaster* because it is easy and inexpensive to maintain this insect model in the lab, the ability to obtain large numbers of individuals to conduct large genome-wide experiments, and the availability of a fully sequenced and annotated genome (Table 1).

**Figure 1:**
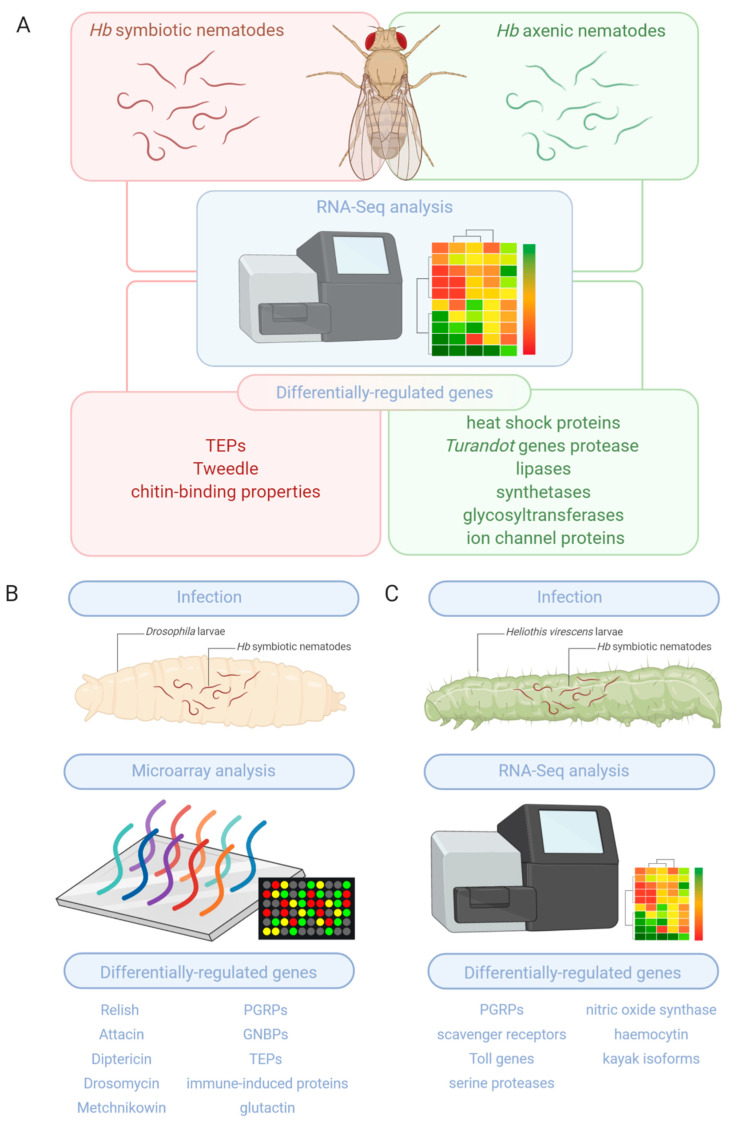
Differentially regulated genes of the insect immune response against entomopathogenic nematodes identified by (**A**) RNA-Seq analysis in *D. melanogaster* adult flies infected by *H. bacteriophora* symbiotic or axenic nematodes; (**B**) Microarray analysis in *D. melanogaster* larvae infected by *H. bacteriophora* symbiotic nematodes; (**C**) RNA-Seq analysis in *H. virescens* caterpillars infected by *H. bacteriophora* symbiotic nematodes.

The first transcriptomic examination of the effect of entomopathogenic nematode infection on gene expression in a model insect host was carried out in *D. melanogaster* [15]. This study employed *D. melanogaster* larvae and the Affymetrix GeneChip microarray system to determine the number and identity of insect host genes, the expression of which is considerably affected following challenge with *Heterorhabditis bacteriophora* symbiotic (carrying their associated *Photorhabdus luminescens* bacteria) nematodes (Figure 1B). The experimental setup was prepared in a way to facilitate the comparison between gene expression changes in uninfected *D. melanogaster* larvae and those infected with symbiotic *H. bacteriophora* that had already released their *P. luminescens* bacteria for at least 6 h after infection. The results from the bioinformatic analyses revealed that 381 *D. melanogaster* larval transcripts were up-regulated and 104 transcripts were down-regulated exclusively upon *H. bacteriophora* infection when compared to transcriptomic data from previous studies involving infection with either bacteria or parasitoid wasps. Further inspection of the results produced a list of the most strongly regulated genes encoding *D. melanogaster* proteins with immune properties [e.g., members of the immune deficiency Imd pathway such as Relish, the antimicrobial peptides Attacin, Diptericin, Drosomycin, Metchnikowin, recognition proteins such as Peptidoglycan Recognition Proteins (PGRP) and Gram-Negative Binding Proteins (GNBP) as well as various immune-induced proteins and thioester containing proteins] [29]. Further, additional genes included those encoding factors that participate in developmental processes and particularly those controlled by the Wnt signaling pathway [30]. Importantly, larvae mutant for the recognition proteins PGRP-LF, GNBP-like 3, the thioester-containing protein 3, and the basement membrane component glutactin exhibited reduced survival ability against *H. bacteriophora*, indicating their participation in controlling entomopathogenic nematode infection in *D. melanogaster*.

Another investigation examined the transcriptomic response of *D. melanogaster* adult flies to *H. bacteriophora* entomopathogenic nematodes [16]. The novelty of this study was that it used for the first time Illumina RNA-Seq technology to interrogate the time-course interaction between symbiotic or axenic (lacking *P. luminescens*) nematodes on *D. melanogaster* physiological processes, including the innate immune response (Figure 1A). An important conclusion derived from this work was that *H. bacteriohora* nematodes, regardless of whether they contain or lack their related mutualistic bacteria, elicit a distinct transcriptomic response in the adult fly compared to infection with *P. luminescens* bacteria alone. Interestingly, the expression of a large number of fly genes was substantially downregulated upon infection with axenic *H. bacteriophora*, suggesting that nematodes deficient of their bacteria are capable of modifying several biological processes in the insect host. Infection of adult *D. melanogaster* with *H. bacteriophora*, and in particular axenic worms, also altered substantially the expression of Heat shock protein and *Turandot* (or *Tot*) genes [31], which indicates elevated levels of stress due to the infection. Similar upregulation was further observed for genes encoding proteins that participate in the regulation of metabolic activities (e.g., protease, lipase, and synthetase enzymes), tissue damage (e.g., glycosyltransferases), and genes involved in nociception (e.g., ion channel proteins). In agreement with the previous transcriptomic study in *D. melanogaster* larvae [15], *H. bacteriophora* symbiotic nematode infection altered the expression of *Tep* genes, which were recently shown to modulate the immune and metabolic response of *D. melanogaster* flies to *Photorhabdus* pathogens [32,33,34,35]; however, their role in the response of the fly to entomopathogenic nematodes is still unknown. Another unexplored question in insect anti-nematode immunity is the nature of proteins that participate in the detection of the parasites. This study reported the upregulation of *Tweedle* genes encoding proteins with chitin-binding properties, which might be involved in nematode recognition in the fly and possibly in other insects.

Transcriptomic responses to entomopathogenic nematodes in model insect hosts have also been expanded to infection by *Steinernema carpocapsae*, which shows increased pathogenicity towards *D. melanogaster* indicating potential differences in gene induction levels compared to *H. bacteriophora* infection [36]. Using an RNA-Seq analysis approach together with a method to generate *S. carpocapsae* axenic nematodes [37], the transcriptomic response of wild-type *D. melanogaster* larvae to this entomopathogenic nematode was tested at two time-points to estimate the time-course changes in gene expression pattern during infection [18]. Bioinformatic analysis showed that although the total number of induced genes at 6 and 24 h post infection was similar, symbiotic *S. carpocapsae* induced slightly higher number of genes than axenic nematodes. Preliminary analysis of these data unveiled that axenic and symbiotic *S. carpocapsae* are able to regulate the expression of multiple *D. melanogaster* genes which are shared between the two types of nematode infections or distinct genes which are specific to infection by one type of nematode. Gene Ontology analysis to determine the nature of the molecular pathways and biological activities they control showed that several immunity-related genes were mostly up-regulated in *D. melanogaster* larvae by symbiotic *S. carpocapsae*, whereas the expression of genes participating in chitin metabolism or neuroactive signaling interactions was mostly affected by axenic nematodes. With regard to immune genes, several genes in the Toll (e.g., *GNBP3*, *Serpin-27A*, and *Drosomycin*) and Imd (e.g., *PGRP-LB*, *Kenny*, *Relish*, and *Attacin*) pathways and fewer genes in the Janus kinase (Jak)-signal transducer and activator of transcription (Stat) (Jak/Stat) (e.g., *Tot-A*) and c-Jun N-terminal kinase (Jnk) (e.g., *puckered*) pathways as well as genes encoding factors that participate in cellular immune activities (e.g., *Tep1*, *Tep2*, and *hemese*) were differentially affected throughout the infection [5,38,39,40]. Interestingly, infection by axenic *S. carpocapsae* in particular also altered the expression of developmental genes in the imaginal disc growth factor family and genes regulated by the Notch and Wnt signaling pathways. 

Understanding gene expression regulation in a natural insect host that is compromised by entomopathogenic nematode infection is important for designing innovative ways to eliminate noxious insect pests. For this, genome-wide changes in the gene expression profile of the tobacco budworm, *Heliothis virescens*, were determined during *H. bacteriophora* infection using Illumina RNA-Seq (Figure 1C) [17]. The approach involved transcriptomic analysis in *H. virescens* larvae at three stages of the infection process: nematode entry into the insect hemolymph, release of symbiotic *P. temperata* bacteria, and nematode encapsulation by hemocytes. Processing the sequencing information identified more than 200 transcripts annotated for immune or stress response. In general, the most striking changes in *H. virescens* gene transcript levels were noted at the nematode invasion and bacterial expulsion. More precisely, a large number of genes was up-regulated during the nematode invasion stage and this pattern was reversed upon release of *P. temperata* with most differentially regulated genes being down-regulated. When the infection was established, although the total number of genes with altered expression was significantly reduced compared to the previous stages of infection, it was observed that the number of up-regulated genes was 3-times more than those down-regulated. Up-regulated genes during the nematode invasion stage included PGRP and scavenger receptors (e.g., *PGRP-2* and *Scavenger receptor class b member 3*), signaling pathway, and response molecules, as well as serine proteases and nitric oxide synthase, (e.g., *Toll*, *Lebocin-4 precursor*, and *Serine protease easter-like*). Down-regulated genes during the release of bacteria by *H. bacteriophora* included some recognition genes (e.g., *Haemocytin*), transcription factors (e.g., *kayak* isoforms), and serine proteases (e.g., *Serine protease snake-like*), whereas during a subsequent phase of infection, the expression of most genes remained unchanged (e.g., *c-type lectin 11*, *Zinc finger protein 691-like*, *Serine protease Persephone-like isoform*, *Moricin-2 precursor*, *Chitinase 5 isoform*). Of note, orthologs of most differentially regulated genes in *H. virescens* are also found in the genomes of other insects, including *Bombyx mori*, *D. melanogaster*, *Tribolium castaneum*, *Aedes aegypt*i, *Culex quinquefasciatus*, *Apis mellifera*, and *Diaphorina citri* [17]. This demonstrates the conservation of genes regulated by entomopathogenic nematode infection the tobacco budworm.

## 3. Mosquito Transcriptomic Response to Filarial Nematodes

Filarial nematodes are important human pathogens because they cause human lymphatic filariasis. They are transmitted by mosquito species in which they develop from microfilariae to infective-stage larvae in approximately one week. Understanding their mode of transmission is paramount for blocking the spread of the parasites to human populations. To achieve this goal, transcriptomic analyses in mosquitoes has already generated important information on the regulation of genes during the development and migration of the nematodes in the infected vector (Table 1).

The mosquito *Armigeres subalbatus* is a vector of the filarial nematode *Brugia malayi* and is capable of activating a distinct immune response against this parasite. In order to understand the details of the time-course molecular events that occur during filarial nematode infection and transmission, expressed sequence tags (ESTs) were generated from cDNA libraries, which were constructed from adult female mosquitoes that had previously been infected with either *B. malayi* through blood feeding or *Dirofilaria immitis* through injection [19]. Following annotation and Gene Ontology analysis, the results showed that several EST clusters encoded immune-related proteins that were subdivided into several categories based on their function. These sub-categories included Caspases, Lectins, Lysozymes, Prophenoloxidases, Thioester-containing proteins, Imd and Toll pathway members, and other unknown molecules, some of which contain homologous sequences in *Anopheles gambiae*, *Ae. Aegypti*, and *C. quinquefasciatus* mosquitoes as well as in the fruit fly *D. melanogaster*. This information is critical for future functional genomic studies to resolve vector competence of mosquitoes for filarial nematodes.

In a more detailed study to determine the specific molecular factors with anti-filarial immune properties in mosquito vectors, the time-course transcriptomic profile of *A. subalbatus* against *B. malayi* was assessed by adopting a natural exposure assay together with microarray analysis [20]. A curious observation was the limited overlap in the type of differentially regulated transcripts between time points. For example, at the early time points, PGRP encoding genes were mostly up-regulated but as the filarial infection progressed, a decrease in the abundance of C-type lectins was found. Because current information on filarial nematode detection in mosquitoes is limited, the observed variation in pattern recognition regulation warrants further attention. Similarly, several transcripts encoding serine proteases (e.g., *Snake-like* and *Easter-like*), which are also found in *D. melanogaster* and potentially take part in anti-filarial immune activities in mosquitoes, were either up- or down-regulated over the time-course of infection. Simultaneously, transcripts encoding antimicrobial peptides with sequence similarity to those found in *A. gambiae* (e.g., *Cecropin*, *Defensin*, *Lysozyme*, and *Gambicin*) were also differentially regulated upon *B. malayi* infection [13]. A novelty of this study was the detection of changes in several transcripts encoding factors with role in cytotoxic reactions (e.g., glutathione synthetase and transferase, cytochrome P450, and peroxidase), which indicates the activation of pathways responsible for metabolizing reactive intermediates upon *B. malayi* infection in *A. subalbatus*. In addition, more than 300 differentially regulated transcripts with no assigned function were described and many of those belong to conserved pathways with a putative immune role. Intriguingly, the type of differentially regulated transcripts in certain mosquito tissues and particularly in hemocytes varied substantially, suggesting a mosquito tissue-specific response against filarial infection.

A similar approach involving DNA microarrays hybridized with RNA probes that were constructed from female mosquitoes was followed to contrast the time-course transcriptome changes in *A. subalbatus* upon bloodmeal infection with either *B. malayi* or *B. pahangi* [21]. Major changes in gene transcript levels in both infection treatments were observed during the first 24 h of infection, which coincides with filarial nematode penetration into the mosquito midgut and invasion of muscle cells in the thorax. Most of the differentially expressed genes had unknown function and only 10% of those genes were related to known or putative immune processes. However, the development of *Brugia* parasites in *A. subalbatus* was accompanied by minor transcriptional changes in the mosquito vector and most differentially regulated transcripts encoded factors irrelevant to immune factors. A characteristic difference in transcriptional regulation between the two infection treatments was the increased number of *A. subalbatus* transcripts encoding reactive metabolites upon infection with *B. malayi*, but not with *B. pahangi* nematodes. This observation might imply a specific protective effect against oxidative damage that occurs by the mosquito melanization response to *B. malayi* parasites.

The transcriptomic response of the mosquito vector to the development of filarial nematodes has further been tested in *Ae. aegypti* during infection with *B. malayi* parasites. Using a genome microarray expression method, the global transcriptomic effects of *B. malayi* on *Ae. aegypti* were assessed at different stages of parasite development [22]. The results revealed a large variation in the number of induced mosquito genes and their level of induction at various developmental stages of the parasite. Similar to *A. subalbatus*, several genes with unknown function were downregulated during the early stages of infection, which correspond to penetration of the midgut and thoracic muscle cells. This illustrates the possibility that filarial nematodes have evolved strategies to counteract the mosquito recognition response upon invasion. When the parasites reach the L1 and subsequently the L2 and L3 stages, the direction of gene expression is reversed from down-regulation to up-regulation and the number of up-regulated genes increases as nematode development progresses. Most of the up-regulated genes encode antimicrobial peptides, mainly cecropins, recognition proteins, and signaling components, serine proteases, and melanization factors, as well as genes with putative immune role. This strongly indicates that development and migration of *B. malayi* in *Ae. agypti* induces a robust immune response.

To dissect the molecular interrelationship between *Ae. aegypti* and *B. malayi*, a dual Illumina RNA-Seq quantitative transcriptome profiling approach was established using tissues from filarial nematode-infected mosquitoes [23]. This study examined the genome-wide temporal gene expression changes that occur at various developmental stages in the parasite and until it reaches the infective stage in the mosquito vector (gene transcription dynamics in filarial nematodes will not be covered in this review). Analysis of the transcriptomic data in the mosquito uncovered two important aspects of the host response to filarial nematode infection. First, *B. malayi* infection over a 6–7-day period alters substantially the expression profile of genes coding for factors that participate in host metabolic activity (e.g., cAMP biosynthesis process, gluconeogenesis and oxidoreductase activity), suggesting a serious metabolic disturbance in *Ae. aegypti* due to parasite development. Second, changes in the expression of glutathione transferases and peroxidases inferred that during filarial nematode infection, the mosquito host is able to suspend toxic derivatives of oxygen metabolism, which forms a mechanism of protection against oxidative damage. Curiously, an *Ae. aegypti* strain with different genetic background was incapable of supporting *B. malayi* development, although the genetic basis of this mosquito-filarial nematode incompatibility was not further determined.

Transcriptomic studies in mosquito vectors have proved useful for the identification of genomic regions that confer resistance to filarial nematodes. An excellent example is described in a study that examines the genetic basis of *Ae. aegypti* immune response to *B. malayi*. [24]. For this, both genomics to identify the *Ae. aegypti* locus responsible for conferring anti-nematode resistance and Illumina RNA-Seq to analyze gene expression changes in resistant and susceptible mosquitos were used. Initial whole-genome sequencing of an *Ae. aegypti* population from Kenya located a single dominant locus and a subset of candidate genes that affect susceptibility to *B. malayi*. Subsequent comparison between the transcriptomic profile of resistant and susceptible *Ae. aegypti* to filarial nematodes during the first 12 h of infection showed strong similarities in the number of differentially regulated genes in the two mosquito populations. Those differentially regulated genes included genes encoding antimicrobial peptides, recognition proteins such as lectins, signaling components in the Imd, Toll, and Jak/Stat signaling pathways, and serine proteases. A small number of genes involved in metabolism, digestion, nutrient transport, egg production, and translation, as well as genes induced through infection by *Wolbachia* were also differentially regulated. Crucially, due to suppressive activity by the parasite or physiological impairment in the host at 48 h post *B. malayi* infection, susceptible mosquitoes were found to down-regulate a group of immune genes encoding antimicrobial peptides and prophenoloxidase. Overall, these results point to the conclusion that resistant *Ae. aegypti* genotypes exhibit enhanced immune response to *B. malayi* nematodes.

The notion that filarial nematodes are able to interfere with immune activities in their mosquito vectors has further been supported by transcriptome microarrays in *C. quinquefasciatus* infected by *Wuchereria bancrofti* [25]. Meta-analysis of the data showed that *W. bancrofti* parasites transmitted by *C. quinquefasciatus* are able to regulate a large number of genes with unknown function and a small number of genes related to immune processes. The latter mainly included genes coding for serine proteases, cuticular proteins, and heat shock proteins. Thus, *W. bancrofti* infection possibly induces melanization, tissue repair, and stress responses in *C. quinquefasciatus*; however, this filarial nematode, like *Brugia* parasites, can develop efficiently in the vector mosquito through evading its immune response.

## 4. Insect Transcriptomic Response to Infection by Parasitic Nematodes

The transcriptomic response of insects to nematode parasites other than entomopathogenic or filarial nematodes is mostly unexplored. The limited number of investigations have concentrated mostly on *Drosophila neotestacea* flies infected by *Howardula* nematodes as well as *B. terrestris* bumblebees and *M. alternatus* beetles infected by *Sphaerularia* and *Bursaphelenchus* nematodes, respectively (Table 1).

Bumblebees *B. terrestris* are naturally infected by the widely distributed nematode parasite *S. bombi*, which confers phenotypic changes to the host, it affects host population dynamics and may enhance the transmission of the parasite. To define the genome-wide transcriptional profile in *B. terrestris* queens upon parasitism by *S. bombi* during overwintering diapause and at later lifecycle stages, a quantitative Illumina RNA-Seq approach was followed [26]. The analysis confirmed that *S. bombi* infection impacts gene expression during *B. terrestris* diapause, but it has a stronger effect on the transcriptional regulation of its host after diapause. Interestingly, distinct sets of genes were differentially expressed in *S. bombi*-infected *B. terrestris* during and after diapause. Differentially expressed genes during diapause were associated with transcriptional and diapause regulation as well as energy metabolism. Those differentially expressed after diapause included genes involved in circadian rhythm, mitochondrial function, and some encoded components of the Toll signaling pathway. Focusing on the transcriptionally affected genes with immune function in *B. terrestris*, those were mostly induced by *S. bombi* after the diapause stage. These included genes encoding immune recognition proteins (e.g., *scavenger receptor*), signaling regulation components (e.g., *domeless*), effector molecules (e.g., thioester-containing proteins), and genes with role in phagocytosis (e.g., *draper*). The outcome from this research suggests that immune activity is modified in *B. terrestris* upon infection with the nematode *S. bombi* and a future challenge will be to determine whether the observed gene expression variation in the infected bumblebees is caused by the parasite per se or it is the result of side effects due to gene dysregulation in the host.

Certain invasive parasitic nematodes are vectored by insects in order to gain access to plants and trees. For example, pine wilt is a disease of pine caused by the pinewood nematode, *B. xylophilus*. The pinewood nematode, which is vectored by the pine sawyer beetle *M. alternatus*, is native to North America and is not considered a primary pathogen of native pines but is the cause of pine wilt in some non-native species [41]. Using global transcriptome information from previous studies that analyzed the response of *M. alternatus* to heat stress and pesticides [27,42], a recent comparative study expanded these findings by probing immune-related gene transcript changes in *M. alternatus* infected by *B. xylophilus* parasites [43]. Using high-throughput Illumina RNA-Seq, it was thoroughly demonstrated that the presence of *B. xylophilus* in *M. alternatus* adults alters the expression profile of a large number of genes with known or putative immune function. Most of these genes were highly conserved in the red flour beetle *T. castaneum* and also showed lower conservation in the mosquitoes *Ae. aegypti* and *A. gambiae*. The conserved genes encoded pathogen recognition proteins (e.g., PGRP, β-glucan recognition proteins, thioester, containing proteins), extracellular signal modulation molecules (e.g., serine proteases and serine protease inhibitors), intracellular signal pathway components (e.g., Toll-like receptors, Spaetzle, MyD88, Relish, Domeless), and various effectors (antimicrobial peptides and prophenoloxidases). This information is pivotal for tackling ecological issues on the association between insects and their natural nematode parasites.

An area of particular interest in the field of insect immunity involves the protective role of endosymbionts against pathogenic infection [44]. With regard to insect parasitic nematodes, *Spiroplasma* endosymbionts have been shown to provide protection to the mushroom-feeding fruit fly *Drosophila neotestacea* upon infection with the natural nematode parasite *H. aoronymphium* [45]. In order to determine the molecular basis of the *Spiroplasma*-modulated priming effect, whole transcriptome Illumina RNA-Seq was performed in *D. neotestacea* flies to identify enhanced immune gene activity that would possibly account for elevated immune response against *H. aoronymphium* infection [28]. Nearly 700 differentially regulated transcripts were detected with more than 300 being up-regulated in response to nematode exposure rather than to *Spiroplasma* infection. Gene Ontology analysis of the immunity-related genes demonstrated that the up-regulated transcripts included some clotting factors (e.g., *fondue*), lectins and proteases, fibrinogen-like domain-containing proteins and molecules regulating chitin metabolism, and a single isoform of the antimicrobial peptide-encoding gene *defensin*. The up-regulated transcripts with Gene Ontology terms for immune response, innate immunity or response to stress were highly conserved in *D. melanogaster* [46]. A few detected down-regulated genes were not related to immune function and mostly encoded proteins participating in translation, cell proliferation, and egg development. Finally, *D. neotestacea* flies contain *Spiroplasma*, a small number of immune genes encoding proteases, and a few prophenoloxidases. Improving defensive symbiosis in insects is an attractive alternative for combating parasitic nematode infection and the information gained from this investigation contributes towards this direction.

## 5. Conclusions and Future Directions

Interactions between nematodes and insects are very common in nature. Nematodes infect a variety of insect species in order to gain access to necessary resources that will allow them to produce their progeny before they move to another suitable host. In addition, nematodes exploit insects for their dispersal and transmission to mammalian hosts where they cause serious infectious diseases. In the case of entomopathogenic nematodes which carry their own species-specific mutualistic bacteria, the interactions implicating the two pathogens and the insect host physiological response are complex and dynamic. To define the kind of interactions that take place at different stages of the infection process and determine whether these interactions impact the insect immune response, the use of molecular and functional techniques has generated exciting information that help us understand the mechanisms involved. An important strategy involves the application of transcriptomic approaches, which have started to decipher the identity of genes in the insect host that participate in the host defense against nematode parasites. Although transcriptomics may have certain limitations such as low resolution, sensitivity and specificity, consideration of partial transcript structure for gene expression, and identification of already recognized genes [47], the use of transcriptomics has revolutionized the field of insect-nematode immunity, because it has started to reveal candidate molecules, the functional role of which can be further confirmed in mechanistic assays. Additionally, a combination of proteomic approaches and systems biology strategies will undoubtedly circumvent the main deficit of transcriptomics, that mRNA transcript levels do not directly correspond to protein levels or function [48]. Future approaches are expected to deploy a combination of transcriptomics and genetic engineering techniques, such as CRISPR gene editing, to unravel the precise role of candidate genes and their products in the interaction of insects with parasitic nematodes.

## Figures and Tables

**Table 1:** Nematodes, their insect hosts, and transcriptomic approaches included in this review.

Nematode Yype	Nematode Species	Insect Host	Transcriptomic Approach	Reference
Entomopathogenic	*Heterorhabditis bacteriophora*	*Drosophila melanogaster*	Affymetrix GeneChip	[15]
	*Heterorhabditis bacteriophora*	*Drosophila melanogaster*	Illumina RNA-Seq	[16]
	*Heterorhabditis bacteriophora*	*Heliothis virescens*	Illumina RNA-Seq	[17]
	*Steinernema carpocapsae*	*Drosophila melanogaster*	Illumina RNA-Seq	[18]
Filarial	*Brugia malayi*	*Armigeres subalbatus*	Expressed Sequence Tags	[19]
	*Dirofilaria immitis*	*Armigeres subalbatus*	Expressed Sequence Tags	[19]
	*Brugia malayi*	*Armigeres subalbatus*	Microarray	[20,21]
	*Brugia pahangi*	*Armigeres subalbatus*	Microarray	[21]
	*Brugia malayi*	*Aedes aegypti*	Microarray	[22]
	*Brugia malayi*	*Aedes aegypti*	RNA-Seq	[23,24]
	*Wuchereria bancrofti*	*Culex quinquefasciatus*	Microarray	[25]
Parasitic	*Shaerularia bombi*	*Bombus terrestris*	RNA-Seq	[26]
	*Bursaphelenchus xylophilus*	*Monochamus alternatus*	RNA-Seq	[27]
	*Howardula aoronymphium*	*Drosophila neotestacea*	RNA-Seq	[28]

## References

[1] Castillo J.C., Reynolds S.E., Eleftherianos I. (2011). Insect immune responses to nematode parasites. Trends Parasitol..

[2] Wang C., Ren M., Liu X., Xia H., Chen K. (2019). Peptidoglycan recognition proteins in insect immunity. Mol. Immunol..

[3] Hillyer J.F. (2016). Insect immunology and hematopoiesis. Dev. Comp. Immunol..

[4] Clark K.D. (2020). Insect Hemolymph Immune Complexes. Subcell. Biochem..

[5] Sheehan G., Garvey A., Croke M., Kavanagh K. (2018). Innate humoral immune defences in mammals and insects: The same, with differences?. Virulence.

[6] Vlisidou I., Wood W. (2015). *Drosophila* blood cells and their role in immune responses. FEBS J..

[7] Wojda I., Staniec B., Sułek M., Kordaczuk J. (2020). The greater wax moth *Galleria mellonella*: Biology and use in immune studies. Pathog. Dis..

[8] Kumar A., Srivastava P., Sirisena P., Dubey S.K., Kumar R., Shrinet J., Sunil S. (2018). Mosquito Innate Immunity. Insects.

[9] Buchon N., Silverman N., Cherry S. (2014). Immunity in *Drosophila melanogaster*—From microbial recognition to whole-organism physiology. Nat. Rev. Immunol..

[10] Getanjaly V.L.R., Sharma P., Kushwaha R. (2015). Beneficial insects and their value to agriculture. Res. J. Agric. Forest. Sci..

[11] Cooper D., Eleftherianos I. (2016). Parasitic Nematode Immunomodulatory Strategies: Recent Advances and Perspectives. Pathogens.

[12] Labaude S., Griffith C.T. (2018). Transmission Success of Entomopathogenic Nematodes Used in Pest Control. Insects.

[13] Famakinde D.O. (2018). Mosquitoes and the Lymphatic Filarial Parasites: Research Trends and Budding Roadmaps to Future Disease Eradication. Trop. Med. Infect. Dis..

[14] Perlman S.J., Spicer G.S., Shoemaker D.D., Jaenike J. (2003). Associations between mycophagous *Drosophila* and their Howardula nematode parasites: A worldwide phylogenetic shuffle. Mol. Ecol..

[15] Arefin B., Kucerova L., Dobes P., Markus R., Strnad H., Wang Z., Hyrsl P., Zurovec M., Theopold U. (2014). Genome-wide transcriptional analysis of *Drosophila* larvae infected by entomopathogenic nematodes shows involvement of complement, recognition and extracellular matrix proteins. J. Innate Immun..

[16] Castillo J.C., Creasy T., Kumari P., Shetty A., Shokal U., Tallon L.J., Eleftherianos I. (2015). *Drosophila* anti-nematode and antibacterial regulators revealed by RNA-Seq. BMC Genom..

[17] An R., Suri K.S., Jurat-Fuentes J.L., Grewal P.S. (2017). Dynamics of transcriptomic response to infection by the nematode *Heterorhabditis bacteriophora* and its bacterial symbiont *Photorhabdus* temperata in *Heliothis virescens* larvae. Insect Mol. Biol..

[18] Yadav S., Daugherty S., Shetty A.C., Eleftherianos I. (2017). RNAseq Analysis of the *Drosophila* Response to the Entomopathogenic Nematode Steinernema. G3.

[19] Mayhew G.F., Bartholomay L.C., Kou H.-Y., Rocheleau T.A., Fuchs J.F., Aliota M.T., Tsao I.-Y., Huang C.-Y., Liu T.-T., Hsiao K.-J. (2007). Construction of an expressed sequences tag library for the mosquito vector *Armigeres subalbatus*. BMC Genom..

[20] Aliota M.T., Fuchs J.F., Mayhew G.F., Che C.-C., Christensen B.M. (2007). Mosquito transcriptome changes and filarial worm resistance in *Armigeres subalbatus*. BMC Genom..

[21] Aliota M.T., Fuchs J.F., Rocheleau T.A., Clark A.K., Hillyer J.F., Chen C.-C., Christensen B.M. (2010). Mosquito transcriptome profiles and filarial worm susceptibility in *Armigeres subalbatus*. PLoS Negl. Trop. Dis..

[22] Erickson S.M., Xi Z., Mayhew G.F., Ramirez J.L., Aliota M.T., Christensen B.M., Dimopoulos G. (2009). Mosquito infection responses to developing filarial worms. PLoS Negl. Trop. Dis..

[23] Choi Y.-J., Aliota M.T., Mayhew G.F., Erickson S.M., Christensen B.M. (2014). Dual RNA-seq of parasite and host reveals gene expression dynamics during filarial worm-mosquito interactions. PLoS Negl. Trop. Dis..

[24] Juneja P., Ariani C.V., Ho Y.S., Akorli J., Palmer W.J., Pain A., Jiggins F.M. (2015). Exome and transcriptome sequencing of *Aedes aegypti* identifies a locus that confers resistance to *Brugia malayi* and alters the immune response. PLoS Pathog..

[25] Bartholomay L.C., Waterhouse R.M., Mayhew G.F., Campbell C.L., Michel K., Zou Z., Ramirez J.L., Das S., Alvarez K., Arensburger P. (2010). Pathogenomics of *Culex quinquefasciatus* and meta-analysis of infection responses to diverse pathogens. Science.

[26] Colgan T.J., Carolan J.C., Sumner S., Blaxter M.L., Brown M.J.F. (2019). Infection by the castrating parasitic nematode *Sphaerularia bombi* changes gene expression in *Bombus terrestris* bumblebee queens. Insect Mol. Biol..

[27] Li H., Zhao X., Qiao H., He X., Tan J., Hao D. (2020). Comparative Transcriptome Analysis of the Heat Stress Response in *Monochamus alternatus* Hope (Coleoptera: Cerambycidae). Front. Physiol..

[28] Hamilton P.T., Leong J.S., Koop B.F., Perlman S.J. (2014). Transcriptional responses in a *Drosophila* defensive symbiosis. Mol. Ecol..

[29] Lemaitre B., Hoffmann J. (2007). The host defense of *Drosophila melanogaster*. Annu. Rev. Immunol..

[30] Bejsovec A. (2013). Wingless/Wnt signaling in *Drosophila*: The pattern and the pathway. Mol. Reprod. Dev..

[31] Ekengren S., Hultmark D. (2001). A family of Turandot-related genes in the humoral stress response of *Drosophila*. Biochem. Biophys. Res. Commun..

[32] Shokal U., Kopydlowski H., Harsh S., Eleftherianos I. (2018). Thioester-Containing Proteins 2 and 4 Affect the Metabolic Activity and Inflammation Response in *Drosophila*. Infect. Immuni..

[33] Shokal U., Eleftherianos I. (2017). The *Drosophila* Thioester containing Protein-4 participates in the induction of the cellular immune response to the pathogen *Photorhabdus*. Dev. Comp. Immunol..

[34] Shokal U., Kopydlowski H., Eleftherianos I. (2017). The distinct function of Tep2 and Tep6 in the immune defense of *Drosophila melanogaster* against the pathogen *Photorhabdus*. Virulence.

[35] Shokal U., Eleftherianos I. (2017). Thioester-Containing Protein-4 Regulates the *Drosophila* Immune Signaling and Function against the Pathogen *Photorhabdus*. J. Innate Immun..

[36] Peña J.M., Carillo M.A., Hallem E.A. (2015). Variation in the susceptibility of *Drosophila* to different entomopathogenic nematodes. Infect. Immun..

[37] Yadav S., Shokal U., Forst S., Eleftherianos I. (2015). An improved method for generating axenic entomopathogenic nematodes. BMC Res. Notes.

[38] Myllymäki H., Rämet M. (2014). JAK/STAT pathway in *Drosophila* immunity. Scand. J. Immunol..

[39] Tafesh-Edwards G., Eleftherianos I. (2020). JNK signaling in *Drosophila* immunity and homeostasis. Immunol. Lett..

[40] Gold K.S., Brückner K. (2015). Macrophages and cellular immunity in *Drosophila melanogaster*. Semin. Immunol..

[41] Zhao L., Mota M., Vieira P., Butcher R.A., Sun J. (2014). Interspecific communication between pinewood nematode, its insect vector, and associated microbes. Trends Parasitol..

[42] Wu S., Zhu X., Liu Z., Shao E., Carballar-Lejarazú R., Guo Y., Xiong Y., Mou Y., Xu R., Hu X. (2016). Identification of Genes Relevant to Pesticides and Biology from Global Transcriptome Data of *Monochamus alternatus* Hope (Coleptera: Cerambycidae) Larvae. PLoS ONE.

[43] Zhou J., Yu H.-Y., Zhang W., Ahmad F., Hu S.-N., Zhao L.-L., Zou Z., Sun J.-H. (2018). Comparative analysis of the *Monochamus alternatus* immune system. Insect Sci..

[44] Eleftherianos I., Atri J., Accetta J., Castillo J.C. (2013). Endosymbiotic bacteria in insects: Guardians of the immune system?. Front. Physiol..

[45] Jaenike J., Unckless R., Cockburn S.N., Boelio L.M., Perlman S.J. (2010). Adaptation via symbiosis: Recent spread of a *Drosophila* defensive symbiont. Science.

[46] Hanson M.A., Hamilton P.T., Perlman S.J. (2016). Immune genes and divergent antimicrobial peptides in flies of the subgenus *Drosophila*. BMC Evol. Biol..

[47] Wang Z., Gerstein M., Snyder M. (2009). RNA-Seq: A revolutionary tool for transcriptomics. Nat. Rev. Genet..

[48] Feder M.E., Walser J.-C. (2005). The biological limitations of transcriptomics in elucidating stress and stress responses. J. Evol. Biol..

